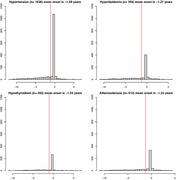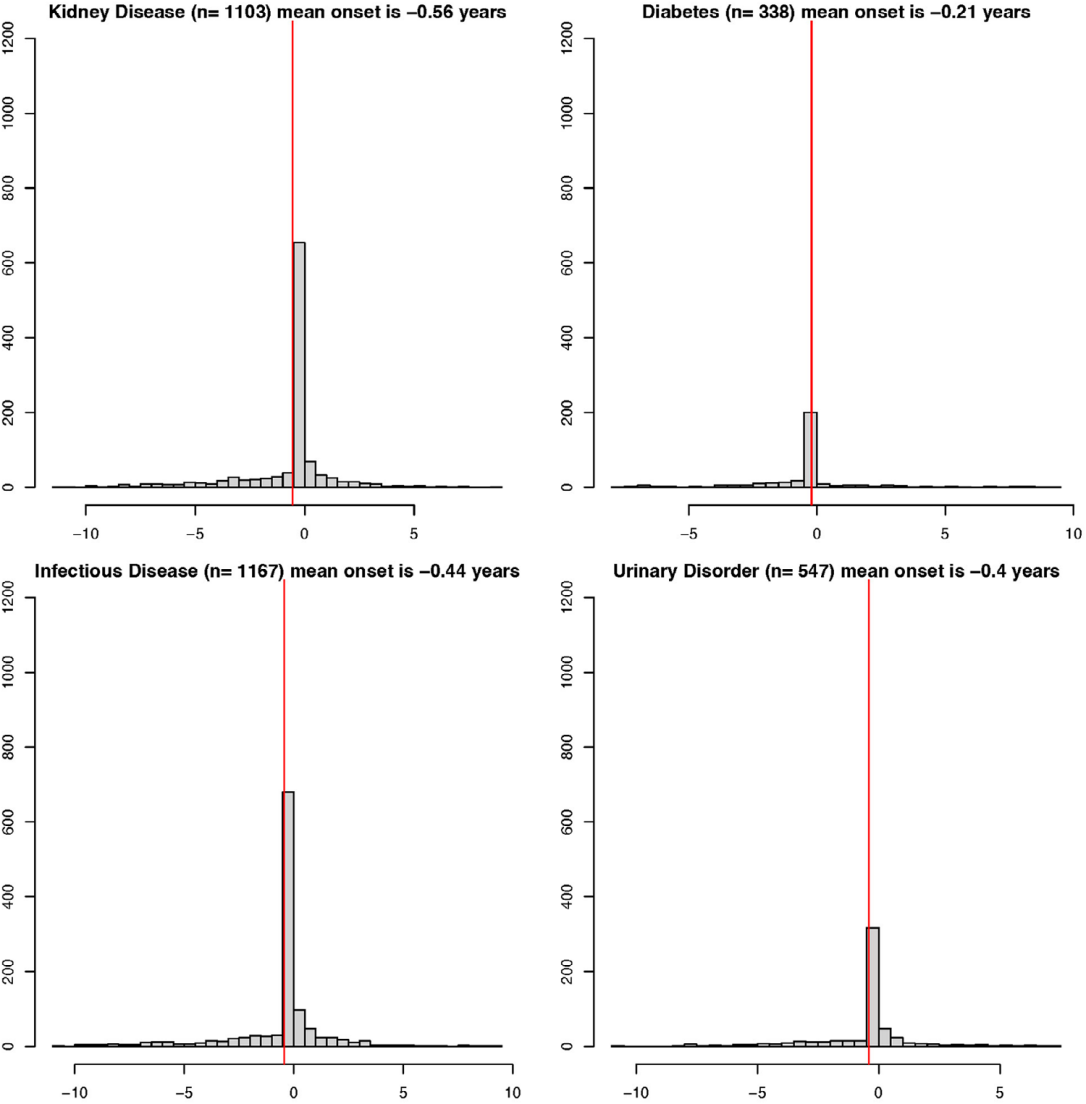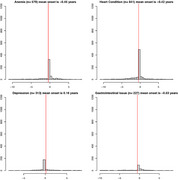# Unraveling Temporal Patterns of Diagnostic Markers and Comorbidities in Alzheimer's Disease: Insights from Large‐Scale Data

**DOI:** 10.1002/alz70856_096810

**Published:** 2025-12-24

**Authors:** Bayard Rogers

**Affiliations:** ^1^ University College London, London, UK, United Kingdom

## Abstract

**Background:**

Comorbid conditions associated with Alzheimer's disease (AD) are poorly understood regarding timing and potential impact on disease onset and progression.

**Method:**

Medical Information Mart for Intensive Care IV electronic health records from 2008 to 2019 were examined. The study identified 2,527 AD patients (34.9% male, mean age 80.27 years) among 299,712 patients. We examined the timing of 12 cardiovascular and metabolic diseases relative to AD diagnosis. Data from the National Alzheimer's Coordinating Center validated the findings.

**Result:**

Hypertension was the most common comorbidity, diagnosed 1.09 years before AD. Depression was the only comorbidity diagnosed after AD start, 0.16 years on average. AD patients had greater rates of hypertension, hypercholesterolemia, and depression compared to the general population.

**Conclusion:**

The findings emphasize early detection and therapy of AD‐related comorbidities, notably cardiovascular and metabolic diseases. The temporal link between these diseases and AD suggests opportunities for preventative strategies and improved care pathways.